# Severe Adaptive Immune Suppression May Be Why Patients With Severe COVID-19 Cannot Be Discharged From the ICU Even After Negative Viral Tests

**DOI:** 10.3389/fimmu.2021.755579

**Published:** 2021-11-19

**Authors:** Yue Zhou, Xuelian Liao, Xiangrong Song, Min He, Fei Xiao, Xiaodong Jin, Xiaoqi Xie, Zhongwei Zhang, Bo Wang, Chenliang Zhou, Yan Kang, Wei Zhang

**Affiliations:** ^1^ Department of Critical Care Medicine, State Key Laboratory of Biotherapy and Cancer Center, West China Hospital, Sichuan University and Collaborative Innovation Center of Biotherapy, Chengdu, China; ^2^ Department of Intensive Care Unit of Gynecology and Obstetrics, West China Second University Hospital, Sichuan University, Chengdu, China; ^3^ Department of Critical Care Medicine, Renmin Hospital of Wuhan University, Wuhan, China

**Keywords:** COVID-19, adaptive immune suppression, secondary infections, transcriptome sequencing, SARS-CoV-2

## Abstract

During the COVID-19 pandemic, a phenomenon emerged in which some patients with severe disease were critically ill and could not be discharged from the ICU even though they exhibited negative viral tests. To explore the underlying mechanism, we collected blood samples from these patients and analyzed the gene expression profiles of peripheral immune cells. We found that all enrolled patients, regardless of changes in genes related to different symptoms and inflammatory responses, showed universally and severely decreased expression of adaptive immunity-related genes, especially those related to T/B cell arms and HLA molecules, and that these patients exhibited long-term secondary infections. In addition, no significant change was found in the expression of classic immunosuppression molecules including PD-1, PD-L1, and CTLA-4, suggesting that the adaptive immune suppression may not be due to the change of these genes. According to the published literatures and our data, this adaptive immunosuppression is likely to be caused by the “dysregulated host response” to severe infection, similar to the immunosuppression that exists in other severely infected patients with sepsis.

## Introduction

Coronavirus disease 2019 (COVID-19), caused by the infection of severe acute respiratory syndrome coronavirus 2 (SARS-CoV-2) (1, 2), has spread throughout the world, causing a devastating medical and social crisis (3, 4). As a highly heterogeneous syndrome, it shows an extensive range of clinical presentations and variable disease progression. Most infected patients are generally asymptomatic or develop mild symptoms (5–8). However, a small proportion (~5%) of patients with COVID-19 progress to a severe condition (6, 9, 10). These patients often require intensive medical treatment because of acute respiratory distress syndrome (ARDS), acute kidney injury (AKI), or multiorgan dysfunction (MODS) with a considerable risk of mortality.

In our clinical practice, we observed that some severe COVID-19 patients with consecutive negative viral tests (SARS-CoV-2 detection by reverse transcriptase polymerase chain reaction of nasopharyngeal swab specimens) remained critically ill and could not be discharged from the ICU. In general, continuous negative viral tests are thought to indicate that the virus has been cleared from the body and that the patients can be considered “recovered”. However, because these patients were still critically ill, they obviously had not truly recovered from the disease. To determine why these patients were still critically ill even though they exhibited negative viral tests, we collected blood samples and analyzed the gene expression profiles of the peripheral immune cells.

We found that all patients, regardless of the changes in genes related to different symptoms and inflammatory responses, showed universally and severely decreased expression of adaptive immunity-related genes, especially those related to T/B cell arms and HLA molecules. This severe adaptive immune suppression may make these patients susceptible to secondary infections. Our data also suggest that this adaptive immune suppression may not be due to the classic immune checkpoint molecules such as PD-1 or long-term use of glucocorticoids but may be caused by the “dysregulated host response” to severe infection, similar to the immunosuppression that exists in other severely infected patients with sepsis.

## Methods

### Study Design

This cross sectional, single center observational study consisted of 14 patients with severe COVID-19 and 5 healthy donors. The patients were enrolled in 2 batches at two different time points (April 2020 and May 2020 separately) in the intensive care unit (ICU) of *East Campus of Renmin Hospital of Wuhan University*, including 5 patients in batch 1 and 9 patients in batch 2. Nasopharyngeal swab specimen which was positive for SARS-CoV-2 by reverse transcriptase polymerase chain reaction (RT-PCR) was used as the diagnosis criteria of COVID-19. In this study, the patients with severe COVID-19 with at least 3 negative virus tests were still in critical ill and could not be discharged from the ICU were enrolled.

### Sample Collection

For each enrolled subject, peripheral venous blood (3mL) was obtained in sodium heparin-coated vacutainers. 3mL Trizol was added directly into each blood sample immediately to inactivate the live SARS-CoV-2 virus and to prevent RNA from degradation. All samples were kept in -80°C until use.

### RNA Sequencing and Data Analysis

Total RNA was extracted from nucleated cells in whole blood. RNA purity was checked using NanoPhotometer spectrophotometer (IMPLEN, CA, USA), and RNA integrity was assessed using the RNA Nano 6000 Kit of the Bioanalyzer 2100 system (Agilent Technologies, CA, USA). A total amount of 1 μg RNA per sample was used as input material for the RNA sample preparations. Sequencing libraries were generated using NEBNext UltraTM RNA Library Prep Kit for Illumina (NEB, USA) following manufacturer’s instructions. The library quality was determined on the Agilent Bioanalyzer 2100 system. Sequencing was performed on an Illumina Novaseq platform. FeatureCounts v1.5.0-p3 was used to count the reads numbers mapped to each gene. Differential expression analysis of two conditions (COVID-19 *versus* healthy) was performed using the DESeq2 R package (1.16.1).

### Statistics

Sequencing data are presented in the form of volcano plots (integrating log2 fold values and multiple-test adjusted probabilities) and heat map plots, generated in R studio and Graphpad prism 8 (GraphPad Software Inc., La Jolla, USA). The resulting P-values were adjusted using the Benjamini and Hochberg’s approach for controlling the false discovery rate. The significance threshold was set to an adjusted P-value <0.05 found by DESeq2. Categorical variables were represented directly as numbers and continuous variables were represented with medians and IQRs. A two-sided P value of < 0.05 was used to indicate statistical significance.

## Results

### Patients Infected With SARS-CoV-2 at Different Periods Have Significant Differences in Genes Controlling Smell and Taste Functions

To obtain a comprehensive understanding of the impact of SARS-CoV-2 infection on patients with severe COVID-19 with at least 3 negative virus tests, we analyzed the transcriptional profiles of whole blood cells *via* RNA-Seq analysis. Five healthy volunteers and 14 patients with severe COVID-19 were enrolled in this study (the characteristics of the patients are listed in **Table 1**). The patients were enrolled in 2 batches at two different time points (April 2020 and May 2020 separately). The first batch (including 5 patients) and the second batch (including 9 patients) both came from the *East Campus of Renmin Hospital of Wuhan University*.

**Table 1:** Demographic and clinical characteristics of the 14 enrolled patients.

Parameter	Patient Batch 1 (N = 5)	Patient Batch 2 (N = 9)
gender (male/female)	3/2	7/2
Age (year)	65 (63, 73)	66 (65, 73)
APACHE II score	–	21 (17, 22)
Mean arterial pressure (mmHg)	91 (79, 105)	75 (65, 99)
Leukocytes (10^9/L)	7.78 (6.04, 8.94)	10.09 (8.78, 14.59)
Neutrophils%	66.5 (65.1, 67.1)	73.6 (66.7, 79.5)
Lymphocytes%	–	12.5 (4.8,15.2)
PLT (10^9/L)	179 (95.75, 265.25)	148 (135, 213)
pH	7.45 (7.41, 7.49)	7.36 (7.31, 7.5)
Lactate (mmol/L)	1.11 (0.65, 1.61)	1.8 (1.2, 2.4)
FiO_2_ (%)	40 (40, 50)	60 (45, 70)
PaO_2_ (mmHg)	123 (110.75, 145.25)	123 (74, 168)
PaCO_2_ (mmHg)	61.2 (56, 62.5)	48 (38, 54)
SpO_2_	98.5 (98, 99.25)	95 (93, 99)
outcome (alive/dead)	4/1	4/5

Values are expressed as medians (interquartile ranges) except sex and outcome. APACHE II score, Acute Physiology and Chronic Health Evaluation II score; FiO_2_, fraction of inspired oxygen; PaO_2_, oxygen partial pressure; PaCO_2_, carbon dioxide partial pressure; SpO_2_, pulse oxygen saturation.

**Figure 1:**
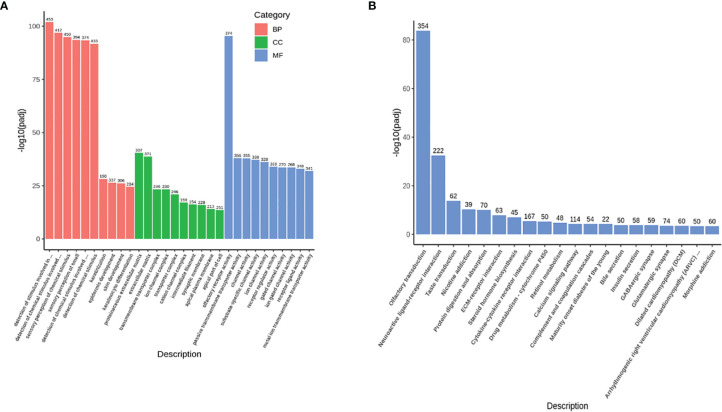
The outline of the RNA-Seq results. **(A)** The GO enrichment items with upregulated genes (33788 genes). The abscissa shows the GO terms, the ordinate shows the significance value (p-adj) of each GO term, and the number at the top of each histogram indicates how many changed genes in each GO term. Different colors represent the three GO subcategories biological process (BP), cellular component (CC), and molecular function (MF). **(B)** The KEGG enrichment items with upregulated genes (33788 genes). The abscissa shows the KEGG terms, the ordinate shows the significance value (p-adj) of each KEGG term, and the number at the top of each histogram indicates how many changed genes in each KEGG term.

**Figure 2:**
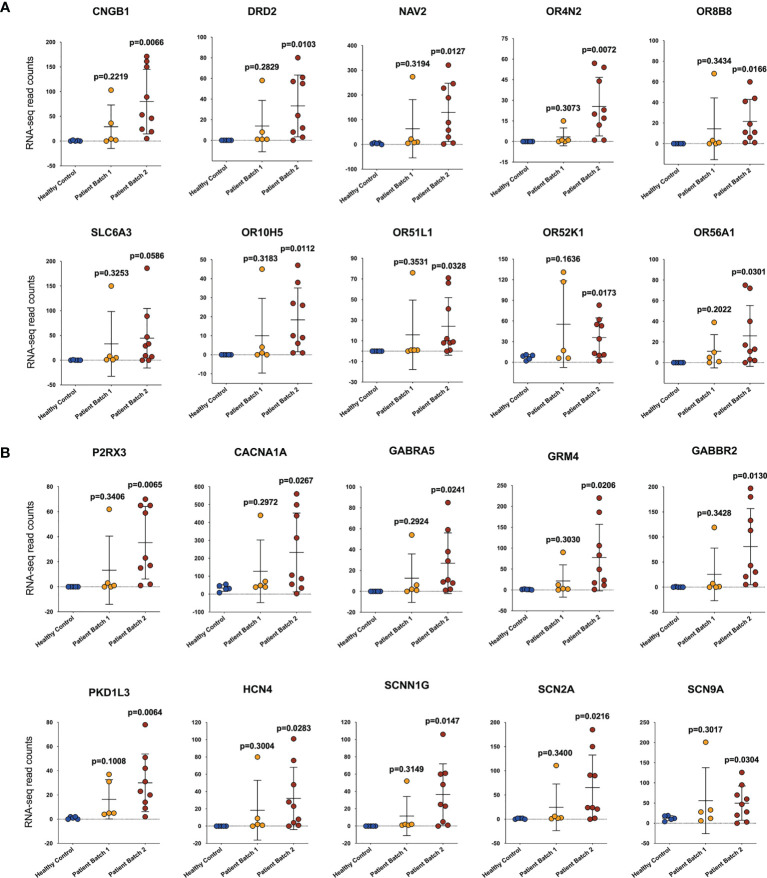
Patients infected with SARS-CoV-2 at different periods have significant differences in genes controlling smell and taste functions. The expression of genes related to smell **(A)** and taste **(B)** function. The abscissa shows the sample source, including healthy controls (HCs) and patients from two batches, and the ordinate shows the gene transcription level by RNA-sequencing read counts. The unpaired, 2-tailed parametric t test with Welch’s correction was used to compare the patient group and the heathy control group. The p values were shown in each panel.

Compared with the healthy donors, the severe COVID-19 patients exhibited 33788 upregulated genes, 1007 downregulated genes, and 20347 genes that remained unchanged. A volcano map (**Supplementary Figure 1**) was used to visually display the differentially expressed gene (DEG) distribution between the patients and the healthy donors. We used Gene Ontology (GO) and Kyoto Encyclopedia of Genes and Genomes (KEGG) analyses to investigate gene enrichment. The most significantly enriched GO terms were “detection of stimulus involved in sensory perception” and “sensory perception of smell” (**Figure 1A**). The most significantly enriched KEGG terms were “olfactory transduction”, “neuroactive ligand-receptor interaction”, and “taste transduction” (**Figure 1B**). Most of the genes described in those terms overlapped and were classified as genes controlling sensory functions, mainly smell and taste. The expression levels of these genes varied among the different batches of patients. In the first batch of patients, only one patient showed abnormally increased expression of the smell- (**Figure 2A**) and taste-related (**Figure 2B**) genes; however, in the second batch, more than half of the patients showed obviously elevated expression of these genes. The results indicate that patients infected with SARS-CoV-2 at different periods have significantly different symptoms regarding the genes controlling smell and taste functions.

### Changes in the Expression of Genes Related to “Cytokine Storm” and Inflammatory Responses

**Figure 3:**
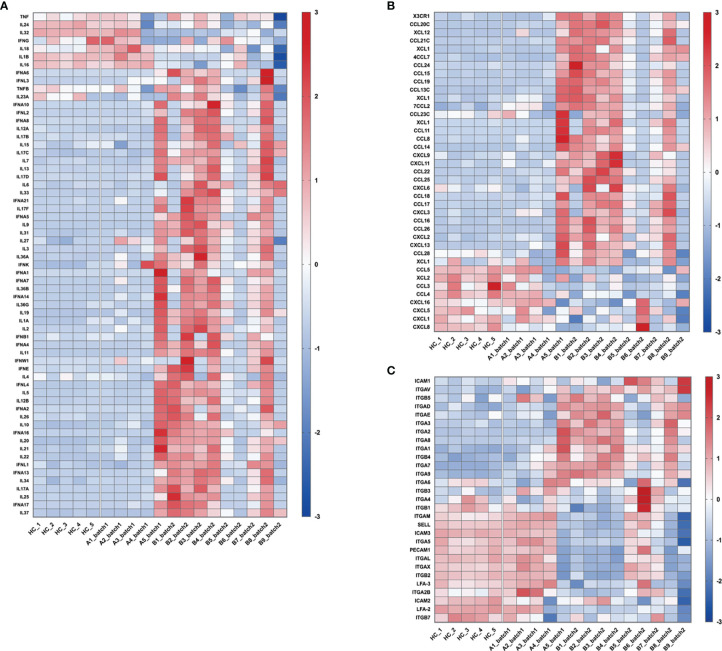
Changes in the expression of genes related to “cytokine storm” and inflammation-related responses. These heatmaps show the relative expression levels of inflammation-related genes: **(A)** cytokines, **(B)** chemokines, and **(C)** adhesive molecules. In the heatmap, each row depicts a different gene, and each column depicts an individual subject, including healthy volunteers and enrolled patients. The relative expression levels of genes were calculated as log_10_FPKM, standardized with the z-score method, and then presented with a pseudocolor scale from -3 to 3. Blue represents downregulation, and red represents upregulation.

Sepsis is defined as life-threatening organ dysfunction caused by a dysregulated host response to infection, including bacterial, fungal, parasitic, or viral infections (The Third International Consensus Definitions for Sepsis and Septic Shock, Sepsis-3) (11). Since SARS-CoV-2 is an infectious viral pathogen, it is reasonable to consider that severe COVID-19 is a subtype of sepsis (12, 13). The “cytokine storm” and inflammatory-related responses are the main focusses of sepsis studies. However, our RNA-Seq results showed that in the patients with severe COVID-19, there were large individual differences in the expression of inflammatory factors (**Figure 3A**), chemokines (**Figure 3B**), and adhesive molecules (**Figure 3C**). Patient A5 from batch 1 and patients B1, B2, B3, B4, and B8 from batch 2 showed significant upregulation of these genes, whereas no obvious changes were found in patients A1, A2, A3, and A4 from batch 1 and patients B5, B6, B7, and B9 from batch 2 (**Figures 3A–C**). In addition, the expression levels of cytokines, chemokines, and adhesive molecules showed no significant difference between the survival and death groups, which is in line with recent COVID-19 studies (14, 15) (**Supplementary Figures 2**–**4**).

### The Adaptive Immune Function of All The Patients Was Severely Impaired

**Figure 4:**
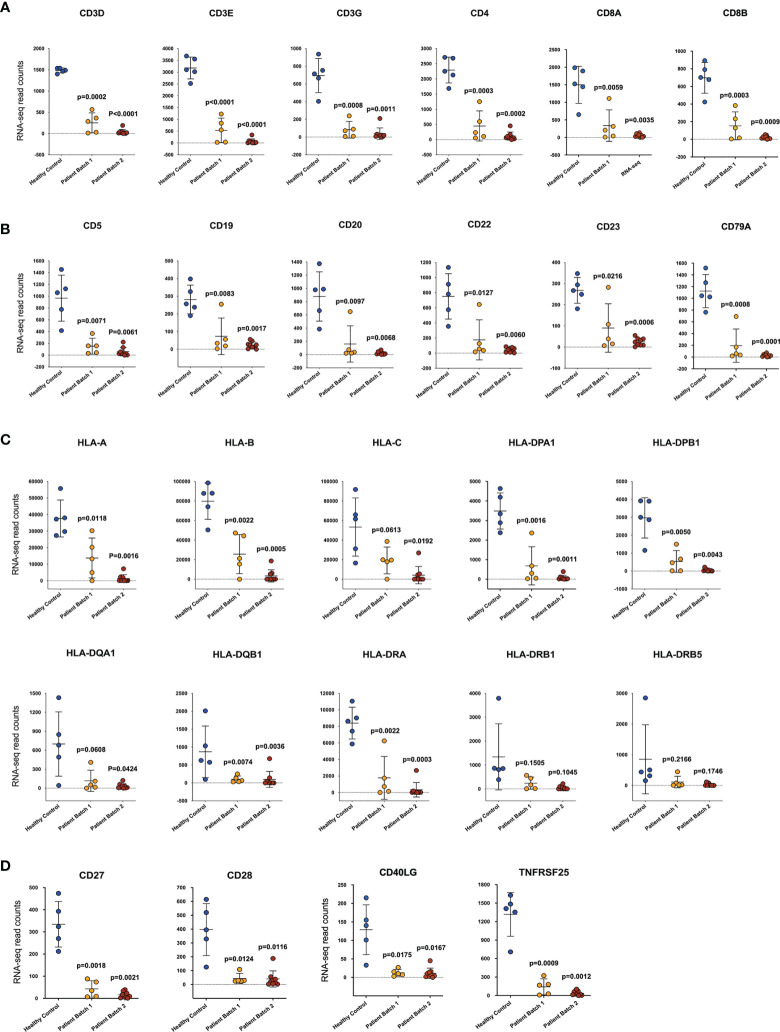
The adaptive immune function of all patients was severely impaired. The transcription level of genes related to T cells **(A)**, B cells **(B)**, HLA molecules **(C)** and costimulatory molecules **(D)**. The abscissa shows the sample source, including healthy controls (HCs) and patients from two batches, and the ordinate shows the gene transcription level by RNA-sequencing read counts. The unpaired, 2-tailed parametric t test with Welch’s correction was used to compare the patient group and the heathy control group. The p values were shown in each panel.

Compared with the number of upregulated genes (33,788), only 1,007 genes were downregulated. Among them, many genes were related to immune functions. Ten significantly changed KEGG pathways were found (p-adj<0.05), and 4 of them were related to immune responses, namely, “primary immunodeficiency”, “T cell receptor signaling pathway”, “haematopoietic cell lineage”, and “Th1 and Th2 cell differentiation”. Seven genes were shared by these 4 categories: CD3D, CD3E, CD3G, CD4, CD8A, CD8B, and CD40LG. Compared with those in the healthy volunteers, the expression levels of these genes in the patients were dramatically decreased (**Figure 4A**). In addition, genes related to B cell function, including CD5, CD19, CD20, CD21, CD22, CD23, CD79a, and CD79b, were also significantly downregulated (**Figure 4B**).

The major histocompatibility complex (MHC) is a collection of genes that code for MHC molecules found on the surface of all nucleated cells (16, 17). In humans, MHC genes are referred to as human leukocyte antigen (HLA) genes. MHC molecules play an important role in the antigen presentation process, which is a key step in the activation of the adaptive immune response. As shown in **Figure 4C**, the expression of multiple HLA genes, in addition to the most reported HLA-DR gene, was decreased to a very low level.

Antigen-induced signals transferred from antigen-presenting cells by MHC molecules to the T cell receptor (TCR) alone are insufficient to activate T cells. Costimulatory and coinhibitory receptors play a pivotal role in T cell activation or inhibition, as they determine the functional outcome of TCR signaling (18, 19). The expression of four classic costimulatory molecules, namely, CD27, CD28, CD40LG, and TNFRSR25, was reduced to a very low level (**Figure 4D**). However, the expression of other costimulatory molecules (**Supplementary Figure 5A**) as well as coinhibitory molecules (20), such as CTLA-4, PD-1, and PD-L1 (**Supplementary Figure 5B**), did not show a significant increasing or decreasing trend. Together, these results suggest that the adaptive immune response is severely impaired, and it may not be due to the upregulation of classic immune checkpoint molecules such as PD-1.

## Discussion

To investigate why some severe COVID-19 patients with negative virus tests were still critically ill and could not be discharged from the ICU, we analyzed the changes in the transcription level of all genes using whole blood cells. We found that all patients, regardless of the changes in their symptoms and inflammatory responses, showed severely and universally decreased expression of adaptive immunity-related genes, especially those related to T/B cell arms and HLA molecules. The results suggest that those patients were in a state of severe adaptive immunosuppression, which may contribute to their critical illness even though they exhibited negative virus tests.

Long-term severe immunosuppression may make these patients susceptible to secondary infections (21). In fact, among the 14 enrolled patients, 5 were confirmed to have secondary infections, as evidenced by laboratory tests, including bloodstream, urinary tract, and multisite infections. Although other patients had no direct laboratory evidence of secondary infections, they all had clinical symptoms of infections and were treated with broad-spectrum antibiotics or antifungal drugs. Among all the patients, patients A4 and A5 showed the lowest expression of adaptive immunity-related genes. Patient A4 was a 73-year-old male patient who had complex infections, including respiratory carbapenem-resistant Acinetobacter baumannii infection, bloodstream gram-negative bacterial infection, and possible urinary tract infection. The patient finally died despite the use of broad-spectrum antibiotics and other supportive treatments. Patient A5 was a 65-year-old male patient with severe Candida parapsilosis infection. After 6 months of intensive medical treatment, including extracorporeal membrane oxygenation (ECMO) treatment and lung transplantation, the patient finally recovered. Overall, the severe adaptive immunosuppression that occurs after negative virus tests may put the patients at risk of secondary infection and may lead to eventual death. Indeed, even after receiving critical care, including systemic administration of broad-spectrum antibiotics/antifungal drugs, the use of ventilators or ECMO, 6 patients (A4, B3, B4, B5, B6, B9) eventually died during hospitalization.

An outbreak of COVID-19-associated mucormycosis (CAM) has recently been reported. The “syndemic” of rhino-orbito-cerebral mucormycosis infections has arisen, with nearly 9000 cases reported thus far from several states in India (22). Poor control of diabetes mellitus is considered an important predisposing factor for CAM (23, 24). However, not all CAM patients have diabetes (approximately 20% of patients have no history of diabetes), and not all CAMs occur in India (25). A prior history of COVID-19 was present in 37% of patients with mucormycosis developing after an initial recovery (26), which suggests that this “initial recovery” may not be a real cure. These patients who appeared to be cured were likely to have severe adaptive immunosuppression, which makes them vulnerable to severe secondary infections such as mucormycosis. Therefore, those critically ill patients with negative SARS-CoV-2 tests should not be simply regarded as recovered because they may still be in a state of severe immunosuppression and in risk of secondary infection. For these patients, appropriate methods should be used to detect their adaptive immune function, and appropriate immunotherapy that can activate the adaptive immune response should be considered.

It has been reported that some patients are immunosuppressed before infection (27). However, in this study, the enrolled patients did not have a history of hematological tumors or the long-term use of immunosuppressants. Therefore, this adaptive immunosuppression should be regarded as a consequence of SARS-CoV-2 infection rather than a pre-existing immunodeficiency. Additionally, patients A1, B1, B5, B7, and B9 had no history of glucocorticoid therapy during hospitalization, indicating that the immunosuppression identified in these patients might not be attributed to glucocorticoid use. Three patients were at very old ages (A1 is 84 years old female, B5 was an 82 years old male, and B9 was an 90 years old male), therefore, the possibility that the age-related immunosenescence may contribute to the impaired adaptive immune response cannot be ruled out.

In this study, we found that PD-1, PD-L1, and CTLA-4 did not show a significant increasing or decreasing trend, and the expression of these genes was too low to cause such a full range of adaptive immune suppression. Therefore, the adaptive immune suppression might not be due to the elevated expression of classic immune checkpoint molecules such as PD-1, at least in the patients included in this study. COVID-19 can be regarded as a subtype of sepsis caused by a specific pathogen (SARS-CoV-2) (28). Inflammation has always been the focus in the study of sepsis-related immune response. It was reported that four classic inflammatory cytokines, including IL-6, IL-8, IL-10, and MCP-1, were significantly elevated in the cytokine release syndrome (CRS) patients. The plasma from severe COVID-19 patients similarly exhibited increased IL-6, IL-10, and MCP-1 levels, but these levels were not as high as those in patients with CRS from other causes such as sepsis and burns (29). Cheng et al. commented on this study and proposed that the expressions of these factors in COVID-19 were significantly lower than in patients with other infections or burns (30). According to our sequencing data (the expression of IL-8 (CXCL8), IL-10, and MCP-1 (CCL2) were shown in **Supplementary Figures 2**, **3**), the transcription levels of them were similar to that of other inflammatory factors and did not seem to be related to the severity of the disease. As for IL-6, its expression was almost undetectable. We speculate that the difference may be due to that they tested the serum protein levels of these cytokines, while we tested the transcription (mRNA) levels of them. It is possible that these inflammatory factors may have been released in large amounts in the early stage of the disease, but the immune cells may not re-synthesize those factors in the late stage of the disease.

The above-mentioned cytokines, as well as the most studied TNF-α, are all classic inflammatory factors that play important roles in innate immunity. Earlier studies believed that the activation of innate immune response and the subsequent “cytokine storm” are key to the occurrence of sepsis. However, over the past decades, clinical trials targeting those molecules have largely failed to improve clinical outcomes, suggesting that the immune changes caused by sepsis should be re-defined. In recent years, adaptive immunosuppression has begun to be considered a feature of long-term hospitalized patients with sepsis (31–33). For the septic patients, the pathogen type of the initial infection is usually unverifiable, but they still suffer from secondary infections for a long period of time and need to receive a variety of broad-spectrum antibiotics, antifungal drugs, antiviral drugs, or combination therapies (34). The latest definition of sepsis was revised at Sepsis-3 in 2016, in which the pathogenesis of sepsis was modified from “non-specific inflammation” to “dysregulated host response to infection” (11). This dysregulated host response was considered as a complex immune response with the concomitant occurrence of excessive inflammatory response and compensatory anti-inflammatory response syndrome. As a result, most patients with sepsis display signs of profound immunosuppression, which is associated with deleterious consequences (35). Therefore, the severe adaptive immunosuppression found in the critically ill COVID-19 patients may not be a special reaction caused by a specific pathogen such as SARS-CoV-2, but may be caused by the severe infection-related “dysregulated host response”, similar to the immunosuppression that exists in other patients with sepsis.

This brings us back to a more essential question: what is the dysregulated host response and how does it happen? Although Sepsis-3 proposed this concept, the immune blueprint of this “dysregulated host response” has not yet been clearly defined. In our opinion, the adaptive immunosuppression should not be simply regarded as a compensatory response to balance the over-activated innate immune response and hyperinflammation. The main role of the adaptive immune-related molecules identified in this study, including the T/B cell-related molecules and MHC molecules, is to recognize and present a specific antigen and activate specific immune responses against that antigen, rather than regulating the expression of innate immune-related molecules. Therefore, the dramatic decrease in the expression of these molecules is not likely to balance the innate immune response, but more like a response that worsens the dysregulated immune reaction. The severe suppression of adaptive immunity and the over-activation of innate immunity may together constitute this “dysregulated host response”, which ultimately leads to critical infection and sepsis.

The studies on COVID-19 are being updated at a very fast pace. Kalicińska and colleagues recently proposed that immunosuppression may be a hallmark of critical COVID-19 (36). They found that the COVID-19 patients showed marked reductions in leukocytes subpopulations and impaired function of T and NK cells, especially in those critically ill patients. Suarez-de-la-Rica reported that the incidence of secondary infection and antimicrobial resistant infection was very high in critically COVID-19 patients (37). Moser reported that the critical COVID-19 patients increased susceptibility for Candida albicans infection (38). Shi analyzed the PBMCs from COVID-19 patients using single-cell mass cytometry (CyTOF) and found CD4^+^ T-cell depletion, plasma cell expansion, and the reduced antigen presentation capacity in those patients (39). Tian analyzed urine samples from COVID-19 patients using quantitative proteomics and found that immunosuppression and tight junction impairment occur in the early stage of COVID-19 infection (40). Although the immunosuppression-related molecules and analytical methods reported in these studies are different, a consensus is that immunosuppression may play an important role in the process of SARS-CoV-2 infection, especially in those critically ill patients.

## Data Availability Statement

The data presented in the study are deposited in the NCBI/SRA repository, accession number PRJNA779249.

## Ethics Statement

The studies involving human participants were reviewed and approved by Biomedical Research Ethics Committee, West China Hospital, Sichuan University. The patients/participants provided their written informed consent to participate in this study.

## Author Contributions

WZ conceived the initial concept and designed the study. YZ, XL, XS, MH, FX, XJ, XX, ZZ, and BW contributed the clinical data and blood samples. YK helped to analyze the clinical data. CZ and YK offered opinion to improve the study. YZ and WZ wrote the paper. All authors contributed to the article and approved the submitted version.

## Funding

This work was supported by the National Natural Science Foundation of China (81972729 to WZ, 81971811 to FX), and Sichuan Science and Technology Program (2020YFS0203 to WZ, 2021YFS0003 to YK, 2020ZYD008 to XL), and Project of Novel Coronavirus Pneumonia in West China Hospital (HX2019nCoV027 to YK).

## Conflict of Interest

The authors declare that the research was conducted in the absence of any commercial or financial relationships that could be construed as a potential conflict of interest.

## Publisher’s Note

All claims expressed in this article are solely those of the authors and do not necessarily represent those of their affiliated organizations, or those of the publisher, the editors and the reviewers. Any product that may be evaluated in this article, or claim that may be made by its manufacturer, is not guaranteed or endorsed by the publisher.
