# Supplementary material for: Severe Adaptive Immune Suppression May Be Why Patients With Severe COVID-19 Cannot Be Discharged From the ICU Even After Negative Viral Tests
Source: Front Immunol. 2021 Nov 19;12:755579. doi: 10.3389/fimmu.2021.755579 (PMC8640185; doi:10.3389/fimmu.2021.755579)
Supplement: Supplementary file 1 [file Image_1.pdf]

Supplementary Figures

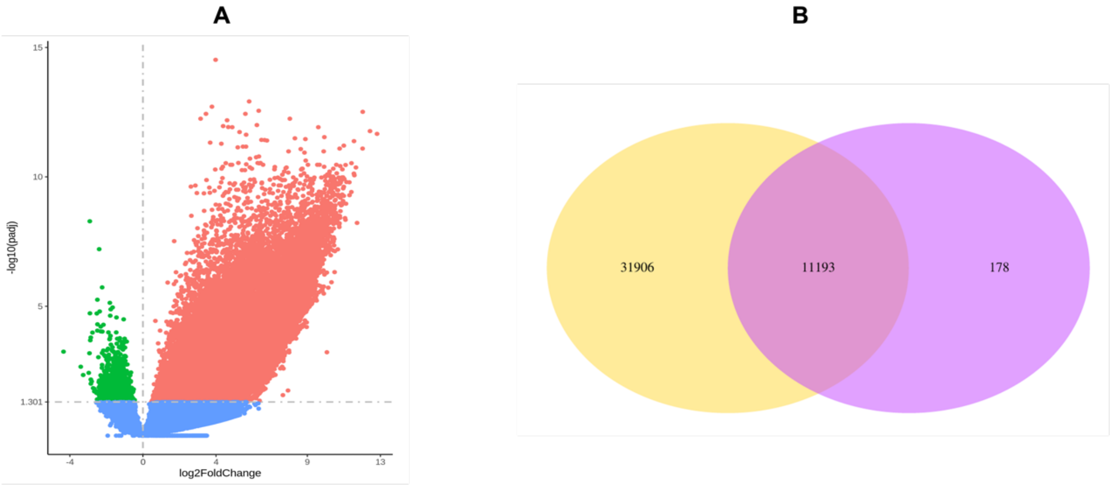

Supplementary Figure 1. The volcano map and Venn diagram show the results of DEGs. (A) The abscissa represents the fold change of gene expression (log_2FoldChange), and the ordinate represents the significance level of the difference in gene expression ($-log_{10} P_{adj}$). Red dots indicate upregulated genes, green dots indicate downregulated genes, and blue dots indicate genes without statistical significance. **(B)** The Venn diagram shows the number of up- and down-regulated genes.

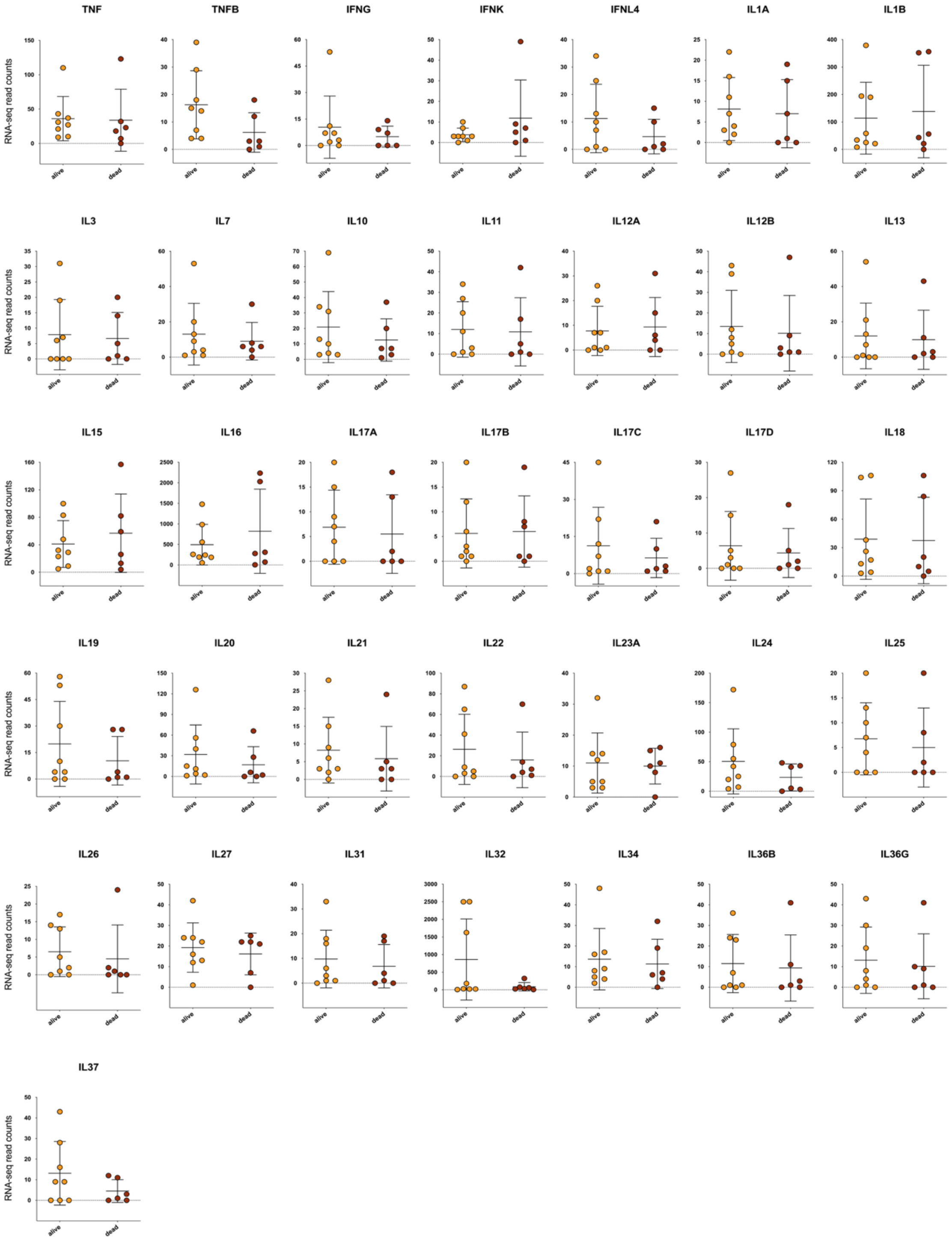

Supplementary Figure 2. The expression level of cytokines showed no significant difference between the survival and death groups. The transcription levels of cytokines with average read counts greater than 5. The abscissa shows the sample source, including live and dead COVID-19 patients, and the ordinate shows the gene transcription level (RNA-sequencing read counts). Significance was assessed

by unpaired t-test with Welch's correction. None of the p-values were < 0.05 (between alive and dead groups).

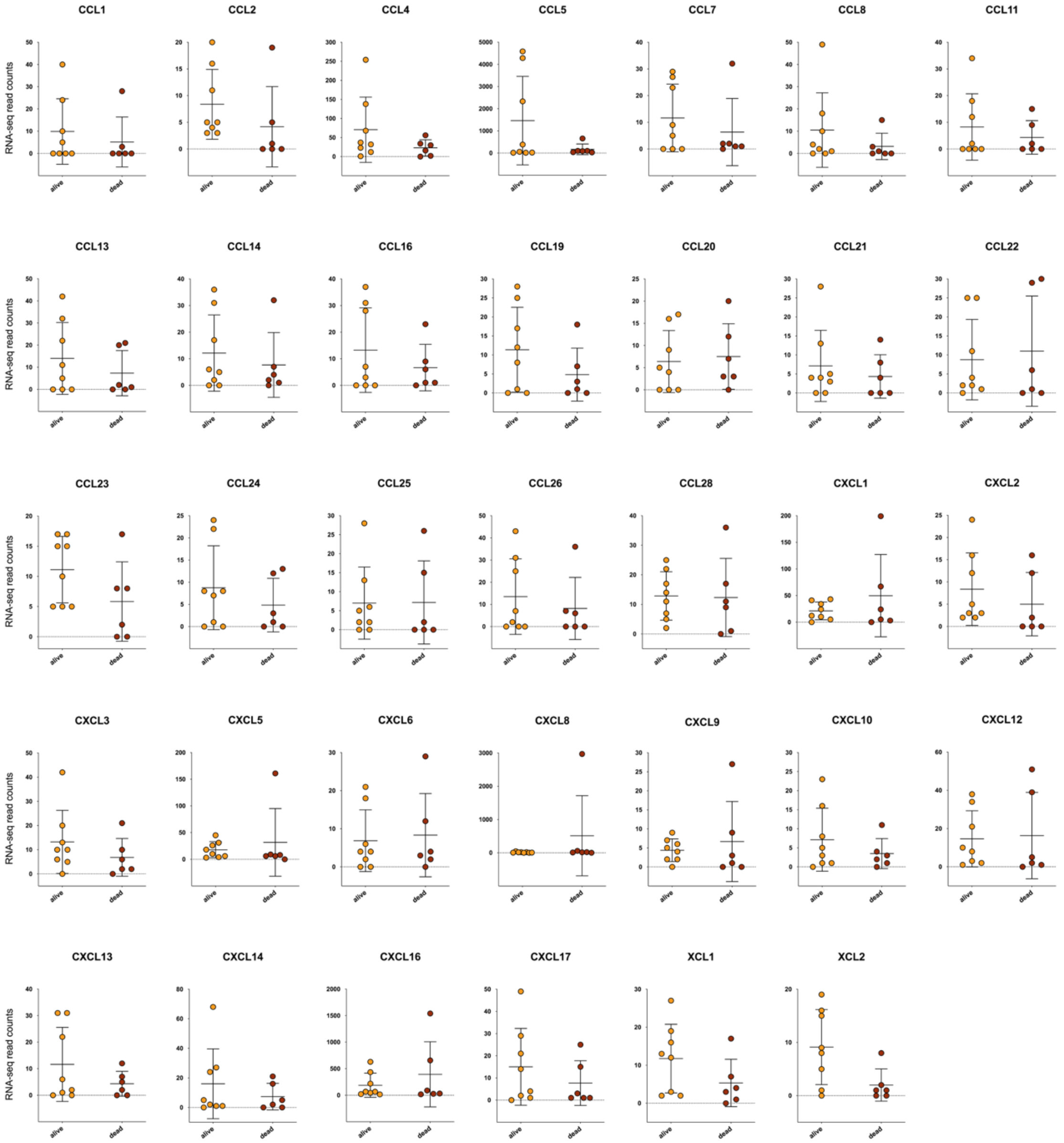

Supplementary Figure 3. The expression level of chemokines showed no significant difference between the survival and death groups. The transcription levels of chemokines with average read counts greater than 5. The abscissa shows the sample source, including live and dead COVID-19 patients, and the ordinate shows the gene transcription level (RNA-sequencing read counts). Significance was assessed by unpaired t-test with Welch's correction. None of the p-values were < 0.05 (between alive and dead groups).

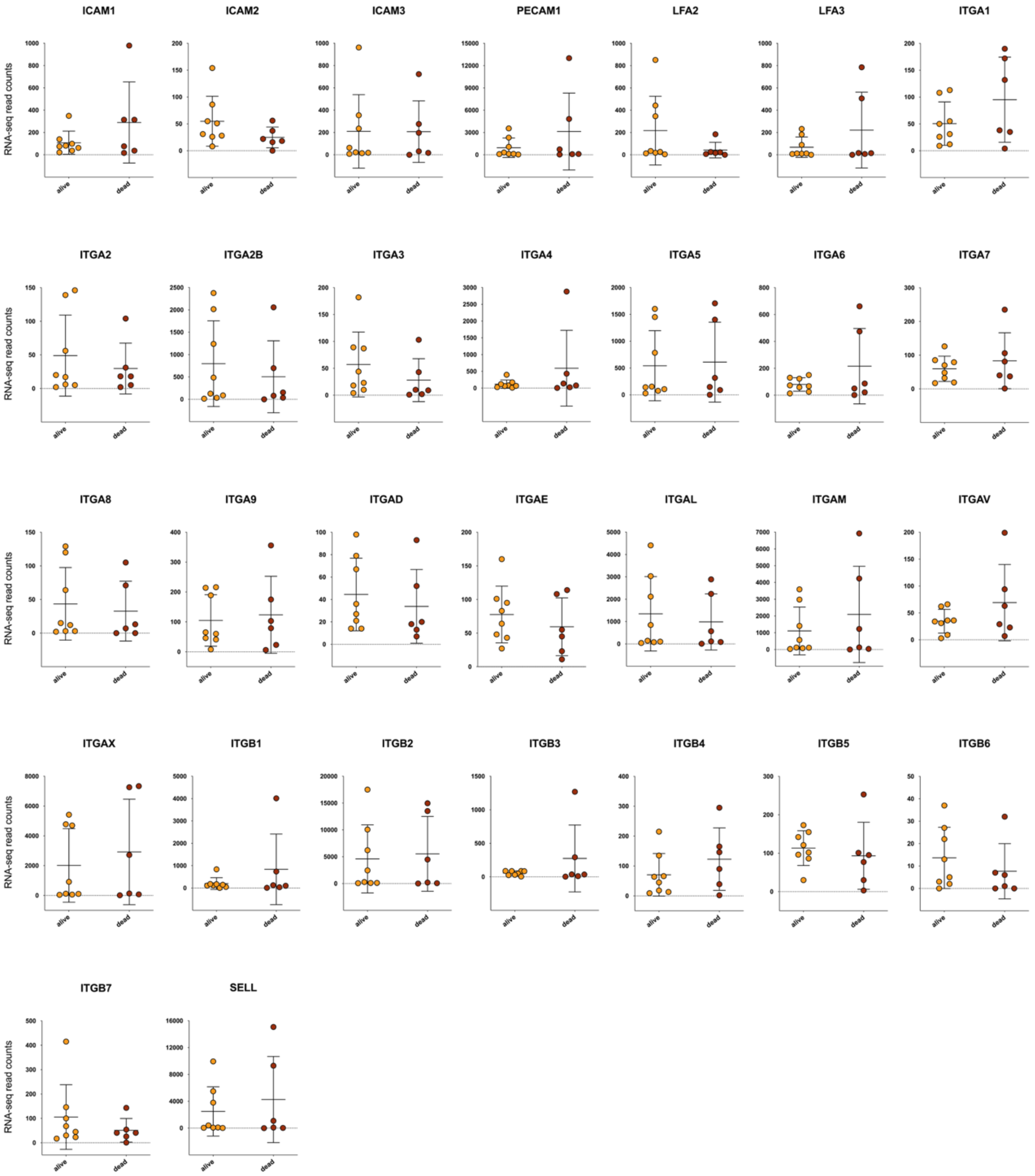

Supplementary Figure 4. The expression level of adhesive molecules showed no significant difference between the survival and death groups. The transcription levels of adhesive molecules with average read counts greater than 5. The abscissa shows the sample source, including live and dead COVID-19 patients, and the ordinate shows the gene transcription level (RNA-sequencing read counts). Significance was assessed by unpaired t-test with Welch's correction. None of the p-values were < 0.05 (between alive and dead groups).

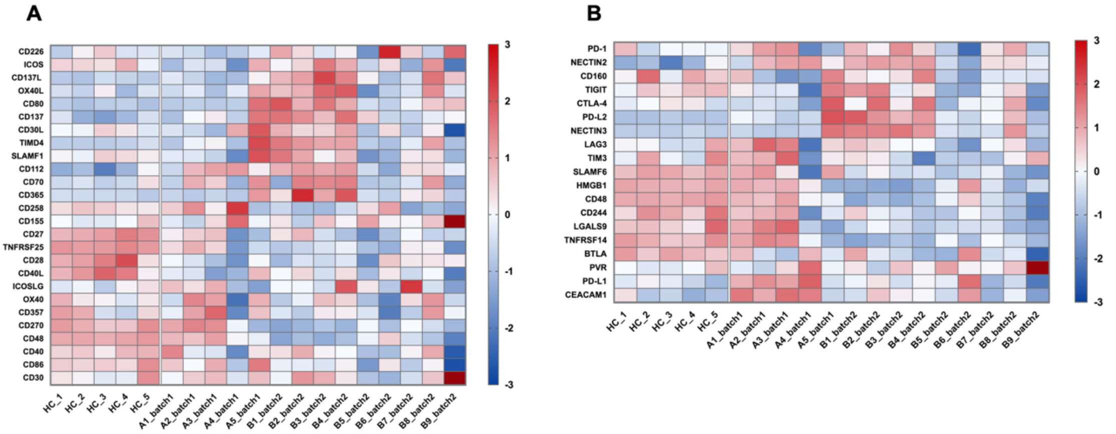

Supplementary Figure 5. The expression levels of costimulatory and coinhibitory molecules. Heatmaps show the relative expression levels of **(A)** costimulatory molecules and **(B)** coinhibitory molecules. In the heatmap, each row depicts a different gene, and each column depicts an individual subject including healthy controls (HCs) and patients from two batches. The relative expression levels of genes were calculated as log_{10}FPKM, standardized with the z-score method, and then presented with a pseudocolour scale from -3 to 3. Blue represents downregulation, and red represents upregulation.
